# Altered Visual Cortical Excitability Is Associated With Psychopathological Symptoms in Major Depressive Disorder

**DOI:** 10.3389/fpsyt.2022.844434

**Published:** 2022-03-07

**Authors:** Hongheng Du, Xue Shen, Xiaoyan Du, Libo Zhao, Wenjun Zhou

**Affiliations:** ^1^Department of Neurology, Yongchuan Hospital of Chongqing Medical University, Chongqing, China; ^2^Division of Clinical Electrophysiology Center, Chongqing Key Laboratory of Cerebrovascular Disease Research, Chongqing, China; ^3^Department of Ophthalmology, Yongchuan Hospital of Chongqing Medical University, Chongqing, China

**Keywords:** major depressive disorder, cortical excitability, visual evoked potentials, paired-pulse suppression, occipital cortex

## Abstract

Previous studies suggest that in people with major depressive disorder (MDD), there exists a perturbation of the normal balance between the excitatory and inhibitory neurotransmitter systems in the visual cortex, indicating the possibility of altered visual cortical excitability. However, investigations into the neural activities of the visual cortex in MDD patients yielded inconsistent findings. The present study aimed to evaluate the visual cortical excitability utilizing a paired-pulse stimulation paradigm in patients with MDD and to access the paired-pulse behavior of recording visual evoked potentials (VEPs) as a marker of MDD. We analyzed the amplitudes of VEPs and paired-pulse suppression (PPS) at four different stimulus onset asynchronies (SOAs) spanning 93 ms to 133 ms. Further, the relationship between PPS and the symptom severity of depression was investigated using Spearman's correlation. We found that, whereas the first VEP amplitude remained unchanged, the second VEP amplitude was significantly higher in the MDD group compared to the healthy controls. As a result, the amplitude ratio (second VEP amplitude/first VEP amplitude) increased, indicating reduced PPS and thus increased excitability in the visual cortex. Moreover, we found the amplitude ratios had a significantly positive correlation with the symptom severity of depression in MDD, indicating a clinically useful biomarker for MDD. Our findings provide new insights into the changes in the excitation-inhibition balance of visual cortex in MDD, which could pave the way for specific clinical interventions.

## Introduction

Major depressive disorder (MDD) is a common mental disease that is characterized by affective disturbances and neurocognitive impairment, for which the development of clinically useful biomarkers remains a challenge (1).

Though the underlying mechanism of MDD has not been fully understood, wide-spread connectivity alterations in the structure and function of cortical regions, including occipital cortical abnormalities linked with impaired visual perception, have been reported in MDD patients (2). Several studies have investigated the cortical processing of different types of visual perceptions in MDD patients, such as visual motion, visual contrast and visual integration, using psychophysical measures. The results revealed higher motion suppression (3), decreased contrast suppression (4) and deficits in integration of visual inputs in MDD (5). The psychophysical deficits in visual perception in MDD are closely related to the abnormality of biochemical changes in the occipital cortex. Using proton magnetic resonance spectroscopy, previous studies have consistently shown a decreased concentration of gamma-aminobutyric acid (GABA) in the visual cortex of MDD subjects (6–8), which can be normalized following effective therapeutic interventions (9–11). As an important inhibitory neurotransmitter, GABA was considered to mediate the center-surround suppression effect in visual perception (12). In addition to GABA, glutamate alterations have been identified in multiple cortical regions, suggesting glutamate has a function in the pathophysiology of MDD as well (13, 14). In healthy individuals, excitatory glutamate levels were found to have a positive association with GABA levels, indicating an excitation-inhibition balance in the occipital cortex. However, in MDD, there was a reduction in glutamate levels in the occipital cortex, and, more importantly, the balance between GABA and glutamate levels was disrupted (8).

Together, these findings suggest that there exists a perturbation of the normal balance between the main excitatory and inhibitory neurotransmitter systems in the occipital cortex of MDD. This begs the issue of whether the excitability of the visual cortex, determined to a great extent by GABA and glutamate, is altered in MDD. In previous studies, transcranial magnetic stimulation (TMS) of the motor cortex revealed that there was an alteration of motor cortical excitability in MDD, which could be modulated by transcranial direct current stimulation (tDCS) (15, 16). The promising intervention of tDCS for treating MDD revealed that aberrant cortical excitability played a significant part in the pathogenesis of MDD. As a result, a greater understanding of the aberrant excitability of the visual cortex may pave the way for new therapy techniques to improve visual perception in MDD patients. So far in the current literature, there are inconsistent findings about alterations involving visual cortical excitability in MDD patients. In an early investigation utilizing electrophysiological measurements, Fotiou et al. revealed that recordings of pattern-reversed visual evoked potentials (VEPs) were within the normal range in MDD patients and were not different from those in healthy controls (17). However, in the two subsequent studies, amplitudes of pattern-reversed VEPs were shown to be considerably lower in MDD patients (18, 19). More recently, Qi et al. explored the relationship between pattern glare and MDD. They discovered a high level of pattern glare in MDD patients, indicating hyper-excitability existed in the visual cortex of MDD patients (20).

Paired-pulse stimulation paradigm, which involves delivering two stimuli at different inter-stimulus intervals, are widely employed to assess cortical excitability. When paired stimuli are applied in close succession, the amplitude of the evoked potential by the second stimulus is suppressed. By comparing the suppressive influence of the second stimulus with the first stimulus, researchers can investigate the cortical excitability in the motor, visual and somatosensory cortex (21–23). High paired-pulse suppression (PPS) indicates low cortical excitability, while low PPS indicates high cortical excitability. To clarify the foregoing seemingly contradictory results, this study employed a paired-pulse stimulation method to produce VEPs. PPS was next examined in MDD patients and a group of healthy controls who were matched by gender, age, and educational level to determine visual cortical excitability. Further, the relationship between visual cortical excitability and psychopathological symptoms in MDD was investigated.

## Methods

### Participants

Twenty-three individuals with MDD and 27 normal controls were enrolled in the study. All subjects were recruited from the neurology outpatient clinics of Yongchuan Hospital and provided written informed consent. This research was carried out in line with the Helsinki Declaration and approved by the Ethics Committee of Yongchuan Hospital of Chongqing Medical University (approval No. 2019114).

The diagnosis of MDD was established by two experienced psychiatrists and confirmed with the Mini International Neuropsychiatric Interview (M.I.N.I.). The population was also assessed psychometrically using the Hamilton Depression Rating Scale (HAMD).

Inclusion criteria of the MDD subjects were: (1) currently in a first or recurrent episode of MDD diagnosed according to the Diagnostic and Statistical Manual of Mental Disorders, Fifth Edition (DSM-V); (2) a total score of HAMD ≥17; (3) age between 18 and 60 years; (4) free from ocular diseases and with normal or corrected-to-normal visual acuity in both eyes; (5) dextromanual and able to finish the study.

Inclusion criteria of the healthy subjects were: (1) no history of psychiatric disorder; (2) a total score of HAMD ≤7; (3) age between 18 and 60 years; (4) free from ocular diseases and with normal or corrected-to-normal visual acuity in both eyes; (5) dextromanual and able to finish the study.

Exclusion criteria for both groups were: (1) history of neurological or other physical illness such as cardiac, respiratory, hepatic, renal, and endocrinal diseases; (2) history or family history of other psychiatric disorders; (3) presence of alcohol or substance abuse; (4) presence of psychotropic drug use; (5) subjects who are pregnant, breastfeeding or in menstrual period.

### Stimulation

The paradigm of paired-pulse stimulation was the same as that used in earlier investigations (24, 25). The participants were situated in a shaded room, 50 cm away from a cathode ray tube (CRT) with a viewing angle of 23° × 17°. The CRT was set to a pixel resolution of 800 × 600 and a frame rate of 75 Hz (13.33 ms per frame). Subjects were instructed to relax with their eyes open and binocularly gaze at a small fixed cross in the center of the monitor. For paired-pulse stimulus, a black and white checkerboard pattern (check size 0.5°, contrast 36 %, mean luminance 16 cd/m^2^) as the initial stimulus was displayed for one frame (13.33 ms), which corresponded to the tube's frame rate. The first stimulus was then followed by presentations of multiple frames with a uniform gray backdrop and no variation in the mean brightness. After various stimulus onset asynchronies (SOAs), the second stimulus, presented as a checkerboard pattern with the same parameters, occurred. We used four different SOAs of 93 ms (six frames), 107 ms (seven frames), 120 ms (eight frames) and 133 ms (nine frames), which had shown paired-pulse inhibition in previous studies. The trials with these paired-pulse stimuli were spaced by 1,000 ms intertrial intervals, resulting in a frequency of around 1 Hz. For single-pulse stimulus, checkerboard patterns with identical contrast and luminance as before were displayed for one frame (13.33 ms), followed by presentations of multiple frames with a uniform gray backdrop (intertrial interval 1,000 ms, resulting in a stimulation frequency of about 1 Hz). Ten trials with paired-pulse stimuli, accompanied by 10 trials with single-pulse stimuli, constituted one cycle. Both the paired- and single-stimulus conditions were given in four successive cycles of ten stimuli, totaling 40 sweeps per condition. The VEPs were performed using a GT-2008V-III VEP system (Guo Te Medical Equipment, Chongqing, China).

### Recording and Analysis

**Figure 1:**
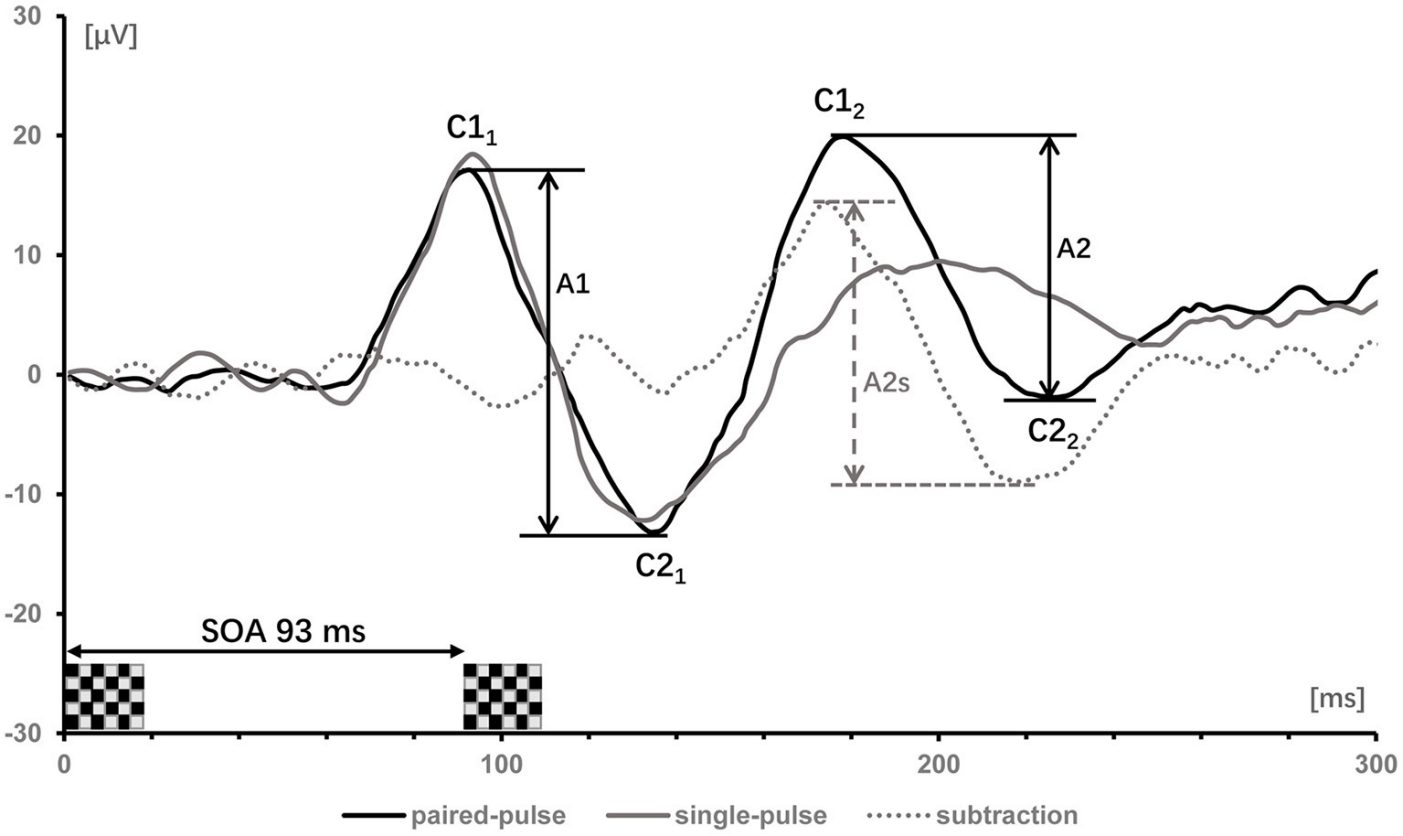
Visually evoked potentials of one subject after single- (gray trace) and paired-pulse with SOA of 93 ms (black trace). The label C denoted the positive and negative components of the first and second response. Subtracting the single-pulse trace from the paired-pulse trace yields the dotted gray trace. The first amplitudes (A1 = C2_1_-C1_1_) and second amplitudes (A2 = C2_2_-C1_2_) in the paired-pulse trace are indicated by vertical bars; the second amplitudes (A2) after subtracting the response of a single-pulse are denoted as A2s.

For VEP recordings, the anodal electrode was inserted on the scalp at Oz (mid-occipital location) above the visual cortex, with the reference electrode at Fz (mid-frontal position) and the ground electrode at Cz (center of the scalp). VEPs of each condition (single stimulation, and paired-pulse stimulation at SOAs of 93, 107, 120 and 133 ms) were documented in epochs from 200 ms before and 300 ms after the stimulation. After being band-pass filtered (1–100 Hz) and baseline adjusted to the Pre-stimulus, signals were averaged and those >140 μV were considered artifacts and were removed. In the single-pulse VEP recordings, C1 denoted a positive peak that occurred <100 ms after stimulus onset, while C2 denoted a negative peak that occurred more than 100 ms after the start of simulus. Considering paired-pulse VEPs, the amplitudes of the first response between C1 and C2 were termed A1 (first amplitude) and the amplitudes of the second response between C1 and C2 were termed A2 (second amplitude). To factored out the linear superposition effects in paired-pulse VEPs, the response to the single-pulse stimulation was subtracted from the paired-pulse stimulation trace, resulting in a “true” second amplitude (A2s). PPS was defined as a ratio (A2s/A1) of the amplitudes of the second (A2s) and the first (A1) peaks (Figure 1). The value of ratio ≥1 means that there is no suppression.

### Statistics

All statistical analysis in this study was done with SPSS (version 19.0). For demographic and clinical data, χ^2^ test and Student's *t* test (unpaired, two-tailed) were conducted to determine the difference between the two groups. For the amplitudes of single VEPs, Student's *t* test was performed. The paired-pulse VEP data were analyzed using a Two-way repeated measures ANOVA (Between-subject factor = group, level = 2; Within-subject factor = SOAs; level = 4) with *post-hoc t*-tests (Bonferroni corrected). To see if there was a link between PPS and HAMD-17 scores, Spearman's correlation coefficient was examined. The general linear regression analysis method is used to analyze the relationships. Differences were considered statistically significant if *p* < 0.05.

## Results

### Demographic and Clinical Characteristics

Table 1 summarizes the demographic variables and clinical characteristics of study participants, including gender, age, education level and the scores of HAMD. No significant differences were observed between the MDD group and the healthy controls with respect to gender (χ^2^ = 0.0480, *p* = 0.8267), age (*T* = 0.5020, *p* = 0.6180) and education level (*T* = 0.1181, *p* = 0.9065). MDD patients had a significantly higher HAMD score than the control group (*T* = 29.7523, *p* < 0.001), showing that the patients were in the midst of a depressive episode when they entered the study.

**Table 1:** Demographic and clinical data of MDDs and healthy controls.

**Variables**	**MDD patients (*N* = 23)**	**Healthy controls** **(*N* = 27)**	**Test statistic**	***p* value**
Gender (M/F)	7/16	9/18	χ^2^ = 0.0480	0.8267
Education, years (SD)	13.7 (1.75)	13.6 (1.80)	*T* = 0.1181	0.9065
Age, years (SD)	30.7 (8.04)	29.7 (5.71)	*T* = 0.5020	0.6180
Age of onset, years (SD)	28.9 (7.30)	–	–	–
Duration of illness, years (SD)	1.7 (1.07)	–	–	–
Number of episodes (SD)	1.2 (0.41)	–	–	–
First/recurrent episode	18/5	–	–	–
HAMD-17 scores (SD)	23.8 (3.4)	3.4 (1.1)	*T* = 29.7523	<0.001

*HAMD, Hamilton Depression Rating Scale. Each value is expressed as mean (standard deviation)*.

### Recording VEPs

Mean values and standard deviations of the response amplitudes to the single- and paired-pulse stimulus in the MDD group and the healthy controls are shown in Table 2. The unpaired Student's *t*-test revealed no significant changes in VEP amplitude between the two groups in the single stimulus condition (*T* = 1.7707, *p* > 0.05).

**Table 2:** Response amplitudes and their ratios for the MDD group and healthy controls.

**Parameter**	**SOAs (ms)**			
	**93**	**107**	**120**	**133**
**MDD group**				
A1 (μV)	34.05 ± 2.04	32.03 ± 2.26	34.24 ± 3.18	32.28 ± 2.14
A2 (μV)	22.64 ± 3.48	26.06 ± 1.83	27.54 ± 3.26	29.10 ± 2.49
A2s (μV)	29.14 ± 2.65	29.28 ± 1.96	30.66 ± 2.25	30.88 ± 2.30
Amplitude ratio (A2s/A1)	0.86 ± 0.08	0.92 ± 0.09	0.90 ± 0.10	0.96 ± 0.10
Single (μV)	32.22 ± 2.68			
**Control group**				
A1 (μV)	32.07 ± 3.10	32.38 ± 2.70	33.24 ± 2.29	32.76 ± 2.43
A2 (μV)	18.77 ± 2.61	20.25 ± 2.60	24.83 ± 3.15	28.08 ± 3.32
A2s (μV)	21.46 ± 1.78	21.86 ± 2.00	25.09 ± 1.71	28.41 ± 2.39
Amplitude ratio (A2s/A1)	0.67 ± 0.07	0.68 ± 0.09	0.76 ± 0.08	0.87 ± 0.10
Single (μV)	33.54 ± 2.59			

*SOAs, Stimulus Onset Asynchronies. Each value is expressed as mean (standard deviation)*.

**Figure 2:**
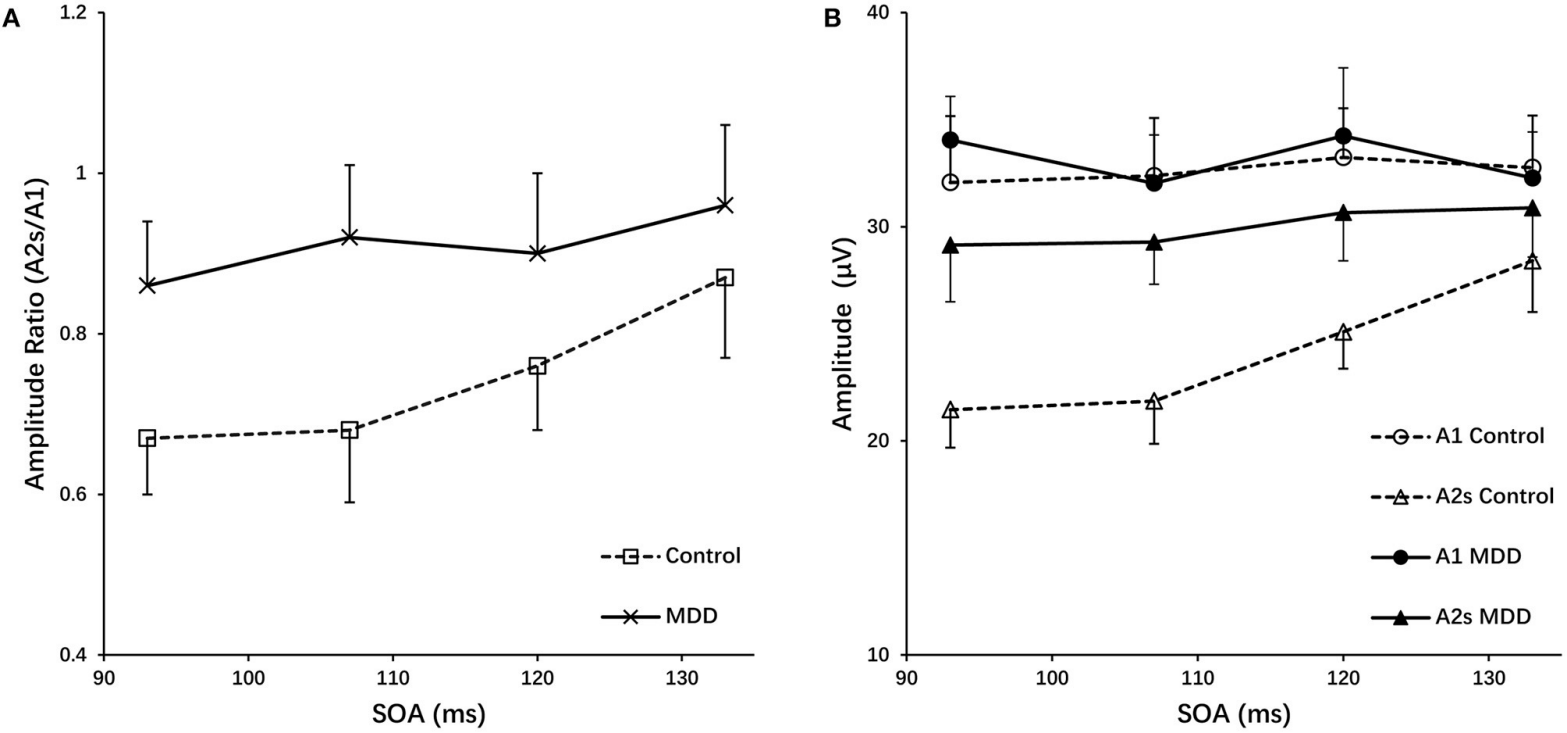
**(A)** Amplitude ratios (A2s/A1) of the MDD and control group as a function of SOAs. **(B)** The first amplitudes (A1) and the second amplitudes after subtracting the response of a single-pulse (A2s) for the MDD and control group vs. SOA, grand mean ± SD.

In the paired-pulse stimulus condition, the amplitude ratios (A2s/A1) in the MDD group and the control group at different SOAs (93 ms, 107 ms, 120 ms and 133 ms) were all <1.0, indicating varying degrees of PPS. The amplitude ratios increased in both groups as the value of SOAs increased, with the largest values (0.96) in the control group at a SOA of 133 ms (Figure 2A). The repeated measures ANOVA for the analysis of amplitude ratio (A2s/A1) indicated there were significant effects of group (MDD vs. control; *F* = 100.467, *p* < 0.001), SOA (*F* = 31.237, *p* < 0.001), and interaction between SOA and group (*F* = 7.451, *p* < 0.001) (Table 3). *Post-hoc t*-tests with Bonferroni correction showed the amplitude ratios (A2s/A1) were significantly higher at SOAs of 93 ms (*F* = 66.481, *p* < 0.001), 107 ms (*F* = 84.846, *p* < 0.001), 120 ms (*F* = 30.536, *p* < 0.001) and 133 ms (*F* = 9.469, *p* = 0.003) in the MDD group compared to the healthy controls (Table 4). A higher amplitude ratio indicated a lower PPS. Because the amplitude ratio was calculated by dividing A2s by A1, the two components were then analyzed separately to identify which component was responsible for the increased amplitude ratio in the MDD group (Figure 2B). For the first VEP amplitude (A1), ANOVA did not indicate any effects of group (MDD vs. control; *F* = 2.592, *p* = 0.114), SOA (*F* = 2.912, *p* = 0.074) or interaction between SOA and group (*F* = 1.824, *p* = 0.156). Regarding the second VEP amplitude after subtraction of the single VEP amplitude (A2s), there were significant effects of group (MDD vs. control; *F* = 256.398, *p* < 0.001), SOA (*F* = 50.096, *p* < 0.001), and interaction between SOA and group (*F* = 17.044, *p* < 0.001). The significant effect of group for A2s indicated that the MDD group had a substantial increase in the second VEP amplitude compared to the control group. *Post-hoc t*-tests with Bonferroni correction showed significantly higher amplitude of A2s at SOAs of 93 ms (*F* = 141.885, *p* < 0.001), 107 ms (*F* = 167.285, *p* < 0.001), 120 ms (*F* = 94.005, *p* < 0.001) and 133 ms (*F* = 13.171, *p* = 0.01) in the MDD group compared to the healthy controls. So, the VEP amplitude to the second stimulus, according to our analyses, is critical in modulating paired-pulse behavior in MDD.

**Table 3:** Effect of SOA, group and their interaction on amplitude ratio (A2s/A1).

**Souce**	**df**	**Observed power**	***p* value**	***F* value**
SOA	3	1.000	<0.001	31.237
Group	1	1.000	<0.001	100.467
SOA*Group	3	0.984	<0.001	7.451

*SOAs, Stimulus Onset Asynchronies; df, degree of freedom*.

**Table 4:** Pairwise comparisons of amplitude ratio (A2s/A1) between MDD and control groups with *post-hoc t*-tests (Bonferroni correction).

**SOAs**	**df**	**Observed power**	***p* value**	***F* value**	**SEM**
93 ms	1	1.000	<0.001	66.481	0.023
107 ms	1	1.000	<0.001	84.846	0.026
120 ms	1	1.000	<0.001	30.536	0.026
133 ms	1	0.854	0.003	9.469	0.029

*SOAs, Stimulus Onset Asynchronies; df, degree of freedom; SEM, Standard Error of Mean*.

### Relation of Paired-Pulse Suppression With Symptom Severity of Depression

**Figure 3:**
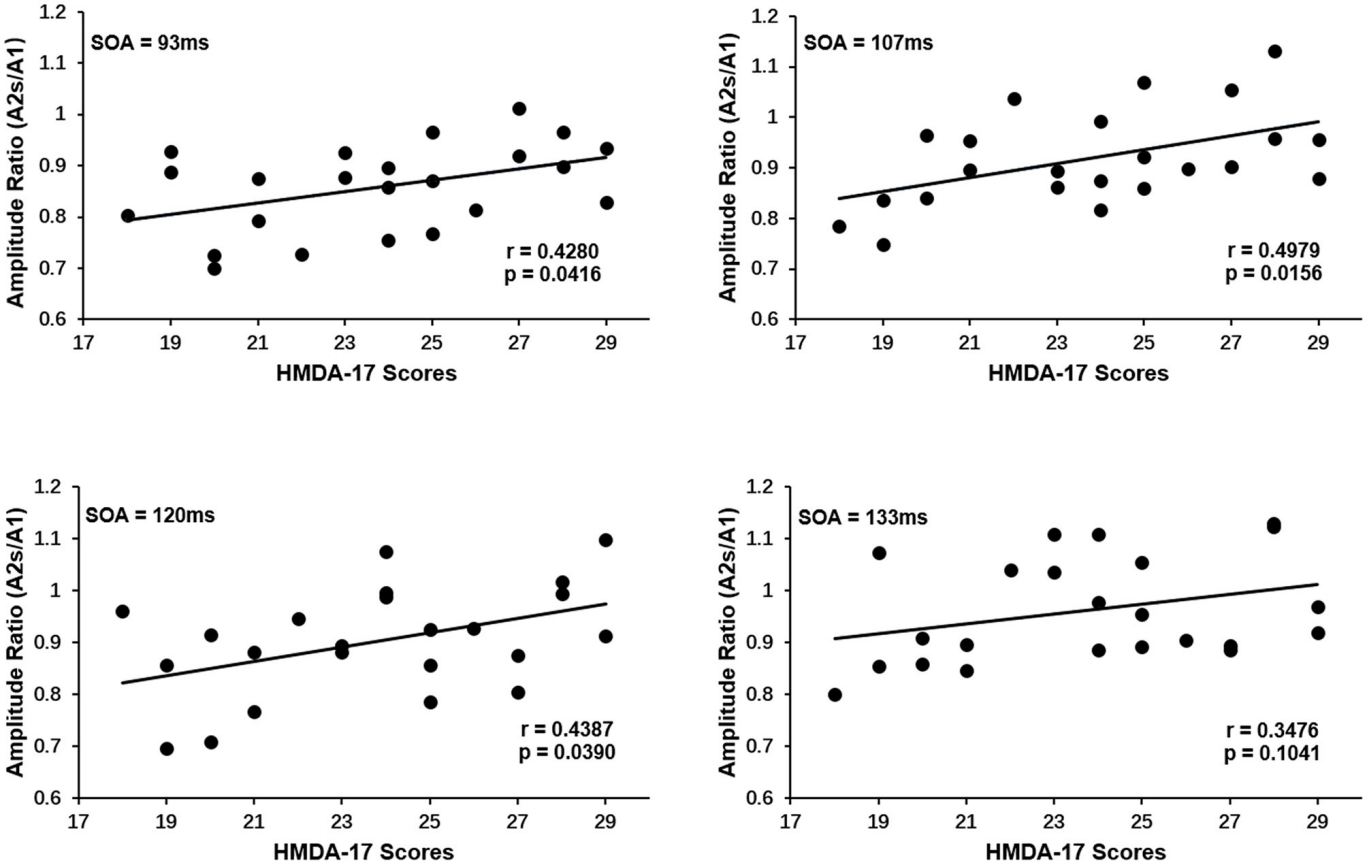
Correlation of paired-pulse suppression with symptom severity of depression for each SOA in MDD group. An increase in the amplitude ratios (A2s/A1) is associated with an increase in HAMD-17 scores. The Spearman's correlation coefficient and p-values for each SOA were present in the lower right corner. Only at SOA of 133 ms was the correlation not significant.

PPS is regarded as a cortical excitability indicator. The above finding that the MDD group showed a reduced PPS and hence an elevated visual cortical excitability begs the question of whether PPS is related to symptom severity in MDD patients. To address this question, a linear association analysis between the degree of PPS and the symptom severity of depression was performed. The amplitude ratios (A2s/A1) used to quantify PPS were shown to have a significantly positive correlation with symptom severity as measured by HAMD scores: the greater the amplitude ratios (A2s/A1), the higher the HAMD scores suggesting higher degrees of symptom severity. Figure 3 showed the Spearman's correlation coefficient and *p*-values for each SOA in the lower right corner of the plots (SOA 93 ms: *r* = 0.4280, *p* = 0.0416, SOA 107 ms: *r* = 0.4979, *p* = 0.0156, SOA 120 ms: *r* = 0.4387, *p* = 0.0390, SOA 133 ms: *r* = 0.3476, *p* = 0.1041). Only at a SOA of 133 ms was the correlation not significant, possibly due to the decayed PPS at long SOAs.

## Discussion

Previous electrophysiologic investigations on neural activities of visual cortex in MDD patients yielded inconsistent results. In our results, we observed no difference in the amplitude of single-pulse VEPs between the MDD group and the healthy controls. This finding is in line with the results reported by Fotiou et al. who found no difference in the amplitude of single VEPs between MDD group and control group, but a significant alteration of VEP latency in different subtypes of MDD (17). However, our finding on single VEP is not in accordance with the two later studies (18, 19), in which amplitudes of pattern-reversed VEPs were shown to decrease significantly in MDD patients. VEPs reflect population synaptic currents and are usually termed for the type of stimulation, such as flash VEPs, pattern-reversal VEPs, or pattern-onset-offset VEPs. The inconsistency of the results might be attributed to the different types of stimulation used in the single VEP recording. Unlike the above mentioned studies, we employed the approach of pattern onset/offset VEPs, which was thought to be less vulnerable to confounding variables including inadequate fixation, eye movements, or willful defocus than pattern reversal VEPs (22). Furthermore, pattern onset/offset VEPs differs markedly from VEPs generated by other forms of stimulation, leading to a new nomenclature known as C1, C2, and C3 (26). The primary source of the C1 component of the pattern onset/offset VEPs is thought to be parvocellular areas of primary visual cortex (V1). The C2 and C3 components appear to be extrastriate in origin (27–29). As a result, comparing multiple research employing different types of stimulation is challenging.

To overcome the challenges of evaluating excitability from single-pulse stimulation, paired-pulse stimulation has become a common method for investigating cortical excitability in various disorders like migraine (30), generalized anxiety (23) or dystonia (31), allowing researchers to better understand about the contributions of inhibition and facilitation in cortex, as well as changes in their balance. We aimed to investigate the visual cortical excitability using a paired-pulse stimulation method in MDD patients, and to access paired-pulse behavior of recording VEPs as a marker of MDD. According to our findings, the amplitude ratios (A2s/A1) were significantly higher in the MDD group compared to the healthy controls, indicating reduced PPS and thus increased excitability in the visual cortex. Further analysis found that, whereas the first VEP amplitude (A1) remained unchanged, the second VEP amplitude (A2s) was significantly higher in MDD patients. Moreover, we found the amplitude ratios (A2s/A1) had a significantly positive correlation with symptom severity of depression, indicating a useful clinical biomarker for MDD.

The result of increased excitability in the visual cortex of MDD patients is consistent with the findings of recent psychophysical research, in which heightened levels of pattern glare were observed in MDD, reflecting an increase in cortical excitability (20). The neurological mechanism driving pattern glare is usually assumed to be of cortical origin, i.e., cortical hyper-excitability or inadequate cortical inhibition caused by a lack of inhibitory systems unable to restrain overexcited situations (32–34). Previous research on cortical excitability in MDD has relied mostly on TMS, which is commonly utilized to evaluate motor cortical excitability, and found an interhemispheric imbalance between the prefrontal and motor cortex, which manifested as decreased excitability in the left hemisphere and increased excitability in the right (35–38). TMS was also utilized in the visual system to test cortical excitability in migraine patients (39), but not in MDD patients. However, TMS can cause phosphene perception in the visual field and incompliance in the subjects when used to measure the excitability of occipital cortex. Hoffken et al. showed that paired-pulse VEPs could indicate equivalent visual cortical excitability features while overcoming the TMS limit, since the PPS of VEPs was inversely linked with TMS-induced phosphene thresholds (40).

PPS, also denoted as forward suppression, refers to the decrease of the neural responding to the second stimuli when two stimuli are presented in short succession. The mechanisms that mediate PPS, on the other hand, are not completely understood. Short-term plasticity, a term used to describe changes in neural behavior resulting from prior activity, is often assumed to represent one possible mechanism, which involves presynaptic depletion of releasable vesicles, postsynaptic receptor desensitization or other presynaptic mechanisms depressing vesicle release (41). In addition, there is evidence for a GABAergic contribution to PPS. GABA is the primary inhibitory neurotransmitter, acting at inhibitory synapses by binding to specific GABA_A_ and GABA_B_ receptors. In rat auditory cortex, research has revealed that forward suppression is primarily regulated by GABA_A_ receptor-mediated inhibition at short ISIs (42). In human motor and somatosensory cortex, drug applications of the GABA_A_ agonist lorazepam could modulate cortical excitability by interfering with GABAergic neurotransmission (43, 44). Moreover, GABA_B_ receptors are also implicated in the regulation of PPS, since presynaptic blockage of GABA_B_ receptors induces a reduction in synaptic release probability, which is compatible with presynaptic inhibition of glutamate release (45). The terms “short-latency intracortical inhibition (SICI)” and “long-interval intracortical inhibition (LICI)” are used in paire-pulse TMS research to describe a phenomenon in which the conditioning stimulus reduces the response of the test stimulus at a short or long ISI, respectively. SICI is supposed to indicate GABA_A_ receptor activity, whereas LICI is thought to reflect GABA_B_ receptor activity. This phenomenon is thought to be related to, and maybe equivalent to, forward suppression (46). In addition to GABAergic systems, glutamate and its receptors were also considered to play an important role in modulating the PPS (47).

PPS can be altered either by changing the response to the first stimulus, or by changing the response magnitude of the second stimulus, which is considered to be controlled by different mechanisms. Modulation in PPS caused by changes in second amplitudes might indicate changes in intracortical processing, whereas the presence of altered first amplitudes reflects an involvement of thalamocortical transmission (30, 48). We observed an elevated change in the second amplitude but no changes in the first amplitude in MDD patients compared to healthy controls, thus reflecting abnormal cortical visual processing in MDD. Indeed, many lines of studies have reported that MDD is often associated with the subjective experience of altered visual perception, such as photophobia, perceived dimness and reduced visual contrast discrimination (49–51). Moreover, the impairment in visual perception was found to be directly related to the psychopathological symptoms of MDD (4, 8). In our study, we found PPS, as indicated by amplitude ratios (A2s/A1), was significantly related to the symptom severity of depression. It is reasonable to presume that aberrant visual cortical excitability has a significant pathophysiological role in MDD, and PPS could serve as a reliable biomarker linking the deficit of visual perception and psychopathological symptoms in MDD.

### Limitations and Alternative Explanations

The paired-pulse stimulation paradigm does not allow for the assessment of separate visual substreams that are differently engaged in visual perception since both the use of a black and white checkboard and a contrast of 36% result in unspecific visual stimulation. The magnocellular pathway is more sensitive to low spatial frequency, low contrast, flicker stimuli and motion detection, whereas the parvocellular pathway is more sensitive to chromatic, high luminance contrast, high spatial frequency and stationary stimuli (52, 53). Given that MDD patients have visual motion perception, visual contrast perception, and visual integration deficits, more research should be done at various luminance contrasts, chromatic colors, and temporal frequencies to separate the possible contributions of parvo-, konio-, and magnocellular streams.

Although our results appear to point to a dysfunctional circuits occurring at the level of visual cortex in MDD, i.e., increased cortical excitability probably due to the imbalance between the excitatory and inhibitory systems, additional possibilities need to be considered. Some neurological disorders are characterized by a defective regulation of contrast gain control, including amblyopia and epilepsy (54, 55). More interestingly, the contrast gain control is likely a property of the transcallosal pathway (56), and also major depressive disorder is characterized by either atrophy or microstructural changes of the corpus callosum (57, 58). In this context, the increased cortical excitability might be attributable to a subcortical impairment in contrast gain control regulation at the corpus callosum.

Another possible explanation for the increased cortical excitability in MDD is the visual cortex's metaplasticity. The presence of mechanisms of metaplasticity could keep synaptic plasticity within a functional dynamic range in the visual cortex, i.e., homeostatic plasticity (59). Many mental diseases are often accompanied by a defective homeostatic plasticity. The elevated change in the second amplitude in MDD patients may be attributed to a deficit of homeostatic plasticity in the visual cortex, which should be explored in future studies.

## Conclusions

In summary, we investigated the paired-pulse behavior of VEPs and found a reduced PPS and thus an increased excitability of the visual cortex in MDD, which may reflect abnormal cortical visual processing. Moreover, PPS had a significant correlation with the symptom severity of depression, indicating a clinically useful biomarker in MDD. Our findings provide new insights into the changes in the excitation-inhibition balance of occipital cortex in MDD.

## Data Availability Statement

The raw data supporting the conclusions of this article will be made available by the authors, without undue reservation.

## Ethics Statement

The studies involving human participants were reviewed and approved by Ethics Committee of Yongchuan Hospital of Chongqing Medical University. The patients/participants provided their written informed consent to participate in this study.

## Author Contributions

HD performed the study, collected data, analyzed data, and drafted the article. XS and XD collected data and analyzed data. LZ designed the research and supervised the project. WZ initiated the study concept, interpreted the data, and revised the article. All authors have read the manuscript and agreed to submit it.

## Conflict of Interest

The authors declare that the research was conducted in the absence of any commercial or financial relationships that could be construed as a potential conflict of interest.

## Publisher's Note

All claims expressed in this article are solely those of the authors and do not necessarily represent those of their affiliated organizations, or those of the publisher, the editors and the reviewers. Any product that may be evaluated in this article, or claim that may be made by its manufacturer, is not guaranteed or endorsed by the publisher.
